# Early High-Dose Erythropoietin and Cognitive Functions of School-Aged Children Born Very Preterm

**DOI:** 10.1001/jamanetworkopen.2024.30043

**Published:** 2024-09-10

**Authors:** Flavia Maria Wehrle, Ulrike Held, Vera Disselhoff, Barbara Schnider, Alexandra Stöckli, Mina Toma, Hans Ulrich Bucher, Jean-Claude Fauchère, Giancarlo Natalucci, Petra Hüppi, Cristina Borradori-Tolsa, Maria Chiara Liverani, Ruth L. O’Gorman, Beatrice Latal, Cornelia Franziska Hagmann

**Affiliations:** 1 Child Development Center, University Children’s Hospital Zurich, Zurich, Switzerland; 2 Department of Neonatology and Intensive Care, University Children’s Hospital Zurich, Zurich, Switzerland; 3 Children’s Research Center, University Children’s Hospital Zurich, Zurich, Switzerland; 4 Department of Biostatistics at Epidemiology, Biostatistics and Prevention Institute, University of Zurich, Zurich, Switzerland; 5 Newborn Research, Department of Neonatology, University Hospital Zurich, Zurich, Switzerland; 6 Family Larsson-Rosenquist Center for Neurodevelopment, Growth and Nutrition of the Newborn, Department of Neonatology, University Hospital Zurich, Zurich, Switzerland; 7 University of Zurich, Zurich, Switzerland; 8 Division of Development and Growth, Department of Woman, Child and Adolescent, University Hospitals of Geneva, Geneva, Switzerland; 9 Center for MR Research, University Children’s Hospital Zurich, Zurich, Switzerland

## Abstract

**Importance:**

Children born very preterm are at risk for long-term neurodevelopmental sequelae. Prophylactic high-dose recombinant human erythropoietin (rhEpo) shortly after birth has not been shown to improve cognitive, motor, and behavioral development at 2 and 5 years.

**Objective:**

To investigate whether early high-dose rhEpo is associated with better executive functions and processing speed—late-maturing cognitive functions—in school-aged children born very preterm.

**Design, Setting, and Participants:**

This single-center cohort study was a prospective, observational follow-up study of a multicenter neonatal clinical trial; 365 children born very preterm (mean gestational age, 29.3 weeks [range, 26.0-31.9 weeks]) who had been enrolled in the Swiss EPO Neuroprotection Trial at birth between 2005 and 2012, and who were included in the primary outcome analyses at 2 years, were eligible to be recruited for the EpoKids study between 2017 and 2021 when they were at school age. Term-born children were additionally recruited and included in a control group. Data were analyzed between May and September 2022.

**Exposure:**

Administration of rhEpo (3000 IU/kg) or placebo (saline, 0.9%) intravenously 3 times within the first 2 days of life as part of the Swiss EPO Neuroprotection Trial.

**Main Outcome and Measures:**

A comprehensive neuropsychological test battery assessed executive functions and processing speed, and parents reported on their child’s executive functions in everyday life to test the hypothesis that early high-dose rhEpo administration is associated with better cognitive outcomes at school age.

**Results:**

In the EpoKids study, 214 children born very preterm (58.6% of 365 children in eligible cohort) were assessed at a mean age of 10.4 years (range, 6.9-13.4 years); 117 (54.7%) were boys. There was no evidence that the 117 children who had received rhEpo differed from the 97 children who had received placebo in any of the 15 executive function and processing speed tests, nor in parent-rated executive functions (estimates ranged from −0.138 to 0.084, all 95% CIs included 0). Irrespective of rhEpo or placebo allocation, children born very preterm scored lower on 11 of 15 executive function and processing speed tests than term-born peers (estimates ranged from 0.112 to 0.255, 95% CIs did not include 0).

**Conclusion and Relevance:**

This study found no evidence for a positive association between prophylactic early high-dose rhEpo administration and long-term neurodevelopmental outcomes after very preterm birth. These results suggest that a comprehensive approach, including pharmacological and nonpharmacological prevention and intervention strategies, is needed to support these children’s neurodevelopmental outcome.

## Key Points

**Question:**

Is high-dose recombinant human erythropoietin (rhEpo) administered to very preterm infants within the first 2 days of life associated with improved cognitive functions at school age?

**Findings:**

In this follow-up cohort study of a neonatal clinical trial including 214 children, the children who had received rhEpo did not show better executive functions and processing speed at school age than the children who had received a placebo.

**Meaning:**

These findings provide no evidence for a positive association between prophylactic early high-dose rhEpo administered to very preterm infants and their long-term cognitive outcome.

## Teaser

This cohort study examines whether early high-dose recombinant human erythropoietin is associated with better executive functions and processing speed among school-aged children who were born very preterm.

## Introduction

Survivors of very preterm birth are at risk for neurodevelopmental problems, including motor, cognitive, and behavioral difficulties.^1,2,3,4,5^ These long-term sequelae are likely a result of the preterm brain’s immaturity at birth and the alterations in structural and functional brain development.^6,7,8^ Neuroprotective interventions aim to improve brain development and, consequently, neurodevelopmental outcome. Among the most promising pharmacological candidates in this regard was erythropoietin due to its neuroprotective properties as shown in experimental models of neonatal brain injury.^9,10,11^ Numerous clinical trials have investigated the effect of erythropoietin on neurodevelopmental outcome of children born very preterm. While a meta-analysis of 5 randomized clinical trials estimated a reduced rate of severe cognitive impairments,^12,13^ neither the large PENUT trial in the United States^14^ nor the Swiss EPO Neuroprotection Trial^15^ found a beneficial effect of prophylactic recombinant human erythropoietin (rhEpo) administered to very preterm infants for their neurodevelopmental outcome at 2 years of age. This was somewhat unexpected as a subgroup of infants in the latter study had undergone cerebral magnetic resonance imaging (MRI) at term-equivalent age and fewer white and gray matter injuries,^16^ and improved white matter development^17^ and structural connectivity^18^ were reported for infants who had received rhEpo compared with those who had received a placebo. Because these improvements in brain development had not translated into better neurodevelopmental outcome, the appropriateness of early childhood assessments for the evaluation of the effectiveness of neuroprotective interventions was called into question. Indeed, brain development continues beyond the neonatal period and beneficial effects may only become apparent later and translate into improved outcome.^19^ This may be particularly true for late-maturing cognitive functions such as executive functions and processing speed that continue to develop throughout childhood, thereby relying on widespread brain networks.^20,21,22,23^

To investigate whether early high-dose rhEpo administration during the first days of life is associated with improved long-term neurodevelopmental outcome, the EpoKids^24^ study followed up children, who had been enrolled in the Swiss EPO Neuroprotection Trial at birth and who were included in the primary outcome analyses at 2 years, when they reached school age. It was hypothesized that those who had received rhEpo would show better executive functions and processing speed compared with those who had received a placebo.

## Methods

### Study Design

This is the prospective, observational follow-up study of a multicenter trial conducted in Switzerland between 2005 and 2012 (Swiss EPO Neuroprotection Trial; NCT00413946). The design of the original trial has previously been described,^15,25^ and the trial protocol is available as a supplement of the primary and secondary outcome reports.^15,16,26^ In short, 448 children born between 26 weeks 0 days and 31 weeks 6 days of gestation were randomized to receive rhEpo (3000 IU/kg) or an equivalent volume of saline placebo (isotonic saline, 0.9%) intravenously at less than 3 hours, 12 to 18 hours, and 36 to 42 hours after birth. The primary outcome of the original trial was the cognitive development quantified as mental development index (MDI) with the Bayley Scales of Infant Development, Second Edition (BSID-II^27^) at 2 years of age.

This prospective, observational follow-up study, EpoKids, assessed the children who had been evaluated at 2 years of age again at school age. Furthermore, term-born children were recruited to allow the comparison of very preterm children with a contemporaneous control group (see Wehrle et al^24^ for the study protocol).

### Participants and Study Procedure

All 365 children born very preterm (mean gestational age, 29.3 weeks [range, 26.0-31.9 weeks]) who had been included in the intention-to-treat analyses of the primary outcome (MDI) at 2 years of age^15^ were eligible for the EpoKids study when they had reached school age. Families were contacted by mail and additionally by phone if they did not reply. A final letter requesting the parents to complete the study questionnaire was sent if families could not be reached. Term-born children were recruited as friends and siblings of the children born very preterm or through advertisement in social media, and at local schools. Exclusion criteria were birth before 37 weeks of gestation, and any parent-reported neurologic or neurodevelopmental disorder (eg, epilepsy, attention-deficit/hyperactivity disorder). Eligibility was verified through a phone screening with a parent.

**Figure 1.**
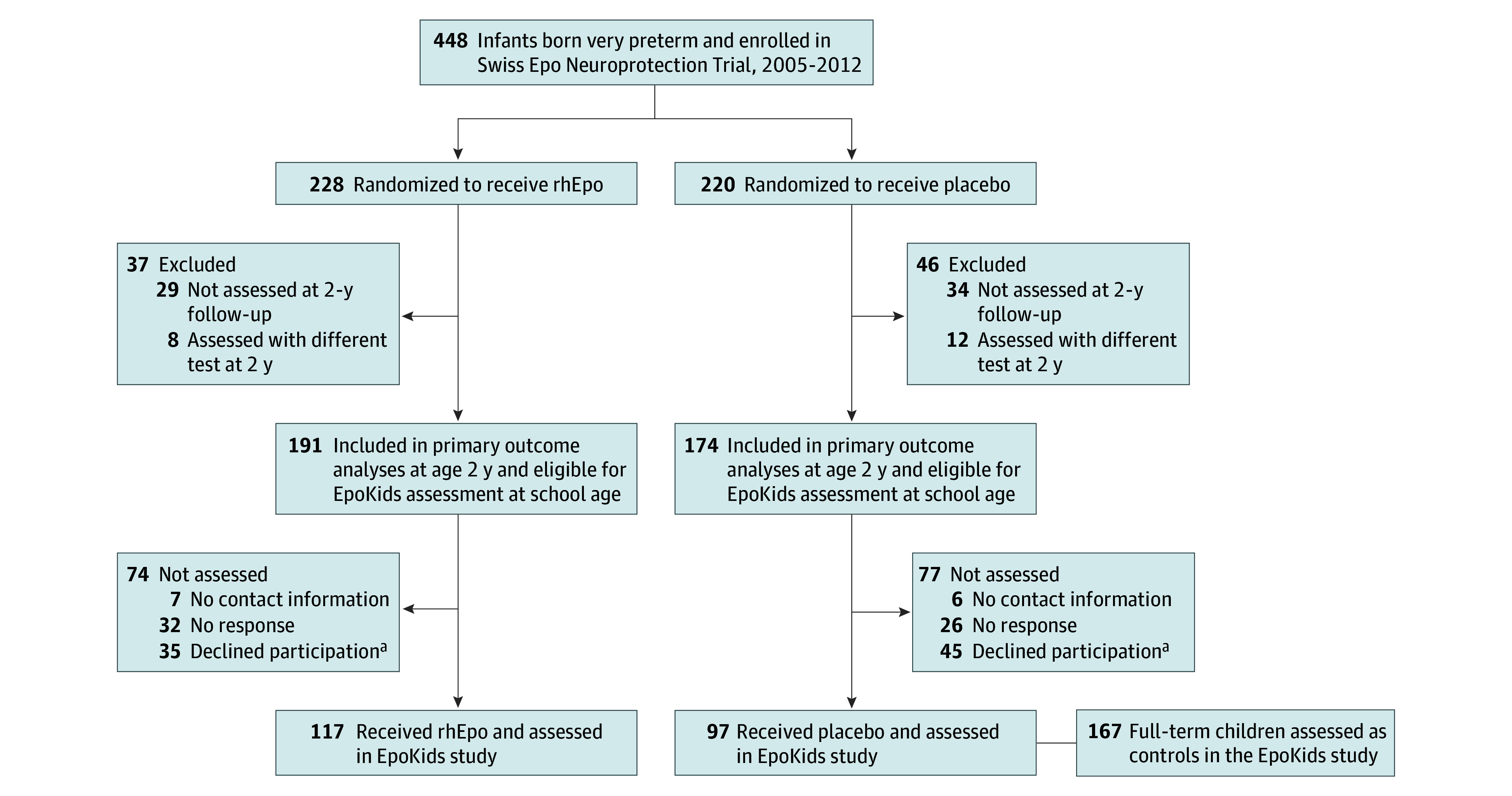
Flowchart of the EpoKids Study The majority of children were assessed at the University Children’s Hospital Zurich, Switzerland, using the German language (n = 162). A small subset of children (n = 12) participated in an abbreviated assessment at the Geneva University Hospitals & University of Geneva, Switzerland, because they lived in close proximity and their first language was French. Only tests for which the impact of language was expected to be minimal (eg, Corsi Block Task) were assessed. Another small subset of children (n = 7) was assessed at home because their families were unable to attend the onsite assessment. For 33 children, parents completed the study questionnaire, including the Behavior Rating Inventory of Executive Functions^35^ but children were not assessed. All 167 term-born children in the control group were assessed in German at the University Children’s Hospital Zurich, Switzerland. rhEpo indicates recombinant human erythropoietin. ^a^Main reasons stated for decline of participation were lack of time, long distance to the assessment site, and generally no interest in study participation.

Children were assessed at the University Children’s Hospital Zurich, Switzerland, the Geneva University Hospitals & University of Geneva, Switzerland, or at home between July 2017 and November 2021 (Figure 1). Examiners were either psychologists and physicians or trained psychology students. All examiners were blinded to treatment allocation in the very preterm group. The full study protocol included a comprehensive neurodevelopmental assessment, a set of self- and parent-reported questionnaires and a 45-minute cerebral MRI. Primary outcomes were executive functions and processing speed, secondary outcomes included academic performance, fine motor abilities, behavioral problems, quality of life, several indicators of the family environment, and brain development (for a full list of assessments refer to the published study protocol^24^). Here, only primary outcomes are reported (ie, executive functions and processing speed). In addition, the estimated IQ (secondary outcome) is reported to describe the overall intellectual functioning of the cohort.

Children received a gift certificate, and travel expenses were reimbursed. The EpoKids study was approved as a prospective, observational cohort study by the ethical committee of the Canton of Zurich, Switzerland. Written informed consent was obtained from parents, and children provided oral consent. Results are reported according to the Strengthening the Reporting of Observational Studies in Epidemiology (STROBE) reporting guideline.

### Outcomes

The EpoKids protocol included instruments that assess key aspects of executive functions and processing speed, both in the verbal and in the spatial domain. They were implemented as either paper-pencil or computerized tests, or they rated executive functions in everyday life. The tests were administered in a randomized order. Inhibition was assessed with Color Word Interference of the Delis-Kaplan Executive Function System (D-KEFS)^28^ and a stop-signal task (Stop-Signal Reaction Time),^29^ previously applied in children born very preterm.^30^ Working memory was assessed with the Working Memory subtest of the Test Battery for Attentional Performance (TAP)^31^, Digit Span of the Wechsler Intelligence Scale for Children, Fourth Edition (WISC-IV),^32^ and the Corsi Block Task.^33^ Cognitive flexibility was assessed with Trail Making (D-KEFS) and the TAP Flexibility subtest. Fluency was assessed with Design Fluency (D-KEFS) and Animal Naming.^34^ Planning was assessed with Tower (D-KEFS). The Behavior Rating Inventory of Executive Functions (BRIEF)^35^ was completed by parents to assess executive functions in everyday life. Processing speed was assessed with Symbol Search and Coding (WISC-IV) and the stop-signal task (No-Signal Reaction Time).^29^ For consistency across all performance-based tests, raw data rather than normative data was used for further analyses, because for some tests, no normative data were available (eg, stop signal task). For parent-rated executive functions in everyday life (ie, BRIEF), T-scores derived from normative data^35^ are presented.

The IQ was estimated with a well-established short-form of the WISC-IV,^32^ including one subtest per index to retain construct validity^36^ and corrected for potential bias of short-form IQ assessments.^37^ Family socioeconomic status (SES) was estimated from parent-reported highest maternal and paternal education, each rated on a 6-point scale (from 0 = no high school qualification to 5 = university degree), and subsequently summed, resulting in a score ranging from 0 to 10 (following Largo et al^38^). If 1 of the 2 education ratings was missing, the other was doubled.

For children born very preterm, perinatal characteristics and the MDI at the 2-year follow-up were retrieved from the database of the original trial.^15^ For term-born children, parents reported on birth weight, gestational age at birth, and on perinatal complications.

### Statistical Analysis

The sociodemographic and perinatal data are displayed as mean and SD, median and IQR, or as number and percentage of total as appropriate. Standardized mean differences (SMDs) address imbalance between children born very preterm who received rhEpo and those who received a placebo, and between children born very preterm and term-born children, with absolute values below 0.1 indicating balance between the groups.^39^

All executive function and processing speed outcomes (eg, number of errors, reaction time) were *z*-transformed and inverted as appropriate to obtain values on the same scale, with higher *z* scores indicating better performance. Multivariable linear regression models were then used to estimate group differences between children born very preterm who had received rhEpo and those who had received a placebo. Separate models were used for each outcome. Adjusted between-group estimates with 95% CIs are reported. Estimates were adjusted for age at assessment, sex, family SES, and gestational age. Interaction effects with intervention group (rhEpo vs placebo) were tested to investigate whether rhEpo moderates the association between any of these variables and executive functions or processing speed. *P* values for interactions were reported without adjustment for multiplicity. The 2 very preterm groups were further compared with the term-born control group, using the same adjustment variables, and the same interactions were tested (except gestational age).

Because there was a considerable amount of missing data across the various outcomes, 50-fold multiple imputation was used to account for the missingness. The missing data–generating mechanism was considered to be missing at random, and the multiple imputation model included all outcomes, the grouping variable (ie, rhEpo, placebo, control group), and the adjustment variables age at assessment, sex, and family SES.

A number of sensitivity analyses were done. First, analyses were repeated with complete cases only. Furthermore, to account for the fact that some families had included more than 1 child in the study and these observations cannot be considered independent, a sensitivity analysis was run, including random intercepts for family. Finally, a per-protocol analysis was conducted, including only those children who had received rhEpo or placebo per protocol.

Details on the sample size calculation for the EpoKids study have been reported previously.^24^ In short, a total of 148 children in each very preterm group (follow-up rate: 81%) was aimed at to detect a minimal effect of *d* = 0.36 with a power of 0.8. An additional 185 children were planned to be included in the term-born control group to allow the potential separate comparison of each very preterm group to a similar-sized control group, given that all eligible children born very preterm (n = 365) were recruited for the EpoKids study.

Data were analyzed between May and September 2022. The statistical programming language R version 4.2.0 (R Project for Statistical Computing) was used,^40^ including the lme4^41^ and mice^42^ package.

## Results

### Study Population

Of the 365 children born very preterm eligible for the EpoKids study, 214 (58.6%) participated (Figure 1). Children born very preterm who participated and those who did not were balanced regarding gestational age (SMD = 0.025). More participants than nonparticipants had received rhEpo (vs placebo, SMD = 0.114), fewer participants than nonparticipants were boys (SMD = 0.252) and participants had a lower birth weight than nonparticipants (SMD = 0.120). Family SES and MDI at 2 years was higher in children who participated compared with those who did not (SMD = 0.503 and SMD = 0.417, respectively) (eTable 1 in Supplement 1).

In EpoKids participants, the 117 children born very preterm who had received rhEpo and those 97 who had received placebo were balanced with regard to sex, family SES, gestational age, and head circumference at birth (all SMD < .100). They were imbalanced regarding birth weight (SMD = 0.116), Apgar at 5 minutes (SMD = 0.326), the 2-year MDI (SMD = 0.145), and age at assessment (SMD = 0.109) (Table 1). Estimated mean (SD) IQ at the EpoKids assessment was not different between the 2 groups (erythropoietin group: 98.5 [10.8] vs placebo group: 100.5 [10.7]; *P* = .32, controlled for age at assessment, sex, family SES, and gestational age).

**Table 1.** Sociodemographic, Perinatal, and Neurodevelopmental Characteristics of Children Born Very Preterm and Typically Developing Term-Born Children Assessed for the EpoKids Study

Characteristics	Erythropoietin group (n = 117)	Placebo group (n = 97)	SMD^a^	Term-born control group (n = 167)	SMD^b^
Sociodemographic					
Sex, No. (%)					
Male	63 (53.8)	54 (55.7)	0.037	83 (49.7)	0.080
Female	54 (46.2)	43 (44.3)		84 (50.3)	
Age at assessment, mean (SD), y	10.3 (1.0)	10.4 (1.1)	0.109	10.7 (1.6)	0.063
Family socioeconomic status, mean (SD)^c^	6.4 (2.4)	6.3 (2.2)	0.064	7.5 (2.1)	0.509
Perinatal					
Gestational age, mean (SD), wk	29.2 (1.7)	29.3 (1.7)	0.099	39.8 (1.1)^d^	7.387
Birth weight, mean (SD), g	1182 (294)	1221 (376)	0.116	3462 (468)^d^	5.569
Head circumference at birth, mean (SD), cm	26.8 (1.9)^e^	27.0 (2.4)^e^	0.080	NA^f^	NA
Apgar at 5 min, median (IQR)	8 (7-9)	8 (6-9)^g^	0.326	NA^f^	NA
2-y Outcome^h^					
Mental Development Index, mean (SD)	95.7 (15.7)	98.1 (16.8)	0.145	NA^i^	NA

Abbreviations: NA, not applicable; SMD, standardized mean difference.

^a^ SMD between the erythropoietin group and the placebo group, with SMD <0.1 indicating balance between groups.

^b^ SMD between the very preterm and the term-born group, with SMD <0.1 indicating balance between groups.

^c^ Estimated from maternal and paternal education, each rated on a 6-point scale and subsequently summed to form a scale ranging from 0 to 10 (higher scores indicate higher family socioeconomic status); for 15 children (8 erythropoietin group, 3 placebo group, 4 term-born control group), data were missing.

^d^ For 1 term-born child, these data were missing.

^e^ For 1 child born very preterm, these data were missing.

^f^ These data were not available for term-born children.

^g^ For 1 child born very preterm, these data were missing.

^h^ Assessed with the Bayley Scales of Infant Development, Second Edition.

^i^ Term-born children were recruited as part of the EpoKids study, thus no 2-year outcome was assessed in these children.

The term-born control group included 167 children. Term-born children came from families with higher SES and, by design, were more mature and heavier at birth than children born very preterm (all SMD > 0.100) (Table 1). Estimated mean (SD) IQ was higher in term-born children compared with children born very preterm (term-born children: 108.8 [10.9] vs children born very preterm: 99.3 [10.7]; *P* < .001, controlled for age at assessment, sex, and family SES).

### Outcome Assessment

**Figure 2.**
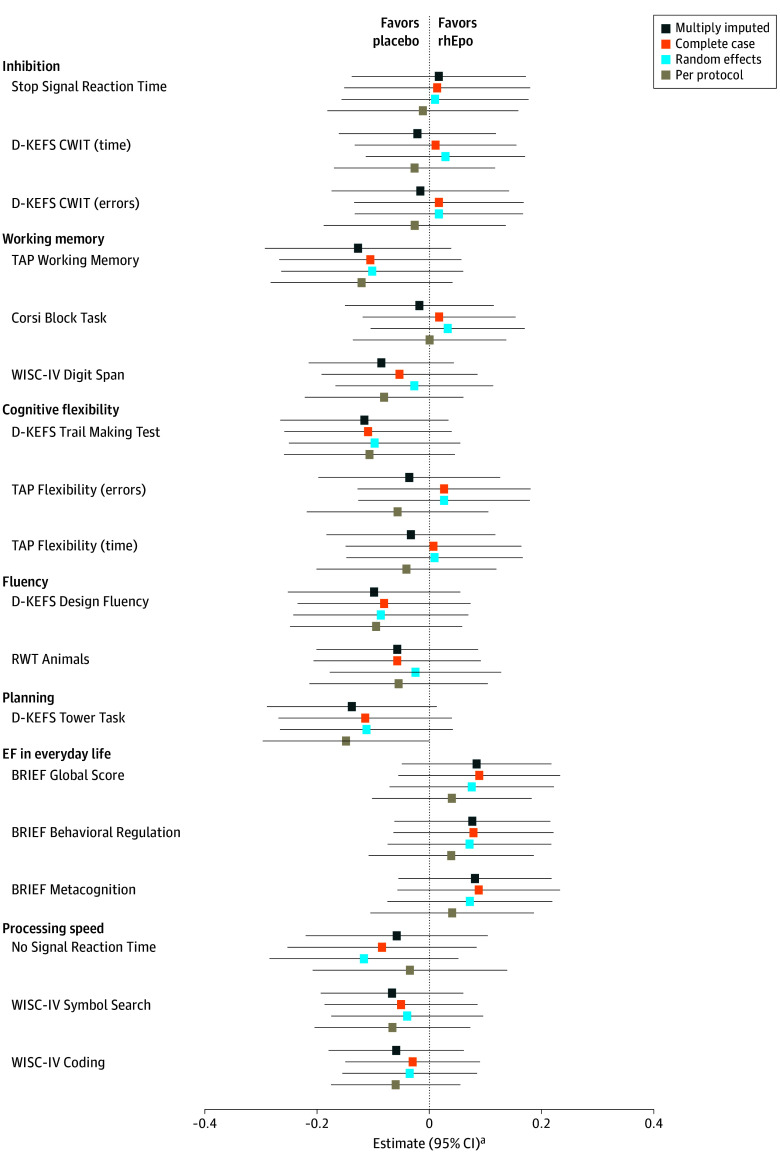
Group Differences in Executive Functions and Processing Speed Between Children Born Very Preterm Who Had Received High-Dose Recombinant Human Erythropoietin Shortly After Birth and Children Who Had Received a Placebo ^a^Coefficients of multivariable linear regression models adjusted for age at assessment, sex, family socioeconomic status and gestational age. BRIEF indicates Behavior Rating Inventory of Executive Functions^35^; CWIT, Color Word Interference Task; D-KEFS, Delis-Kaplan Executive Function System^28^; RWT, Regensburger Verbal Fluency Test (Regensburger Wortflüssigkeitstest)^34^; TAP, Test Battery for Attentional Performance (Testbatterie zur Aufmerksamkeitsprüfung)^31^; WISC-IV, Wechsler Intelligence Scale for Children, Fourth Edition, German version^32^.

Descriptive statistics of executive function and processing speed outcomes are shown in Table 2. Figure 2 illustrates the group differences between children born very preterm who had received rhEpo and those who had received a placebo. There was no evidence of a group difference in any of the outcomes, adjusting for age at assessment, sex, family SES, and gestational age (range of estimates: −0.138 to 0.084, all 95% CIs included 0) (eTable 2 in Supplement 1). None of the interactions were significant (all *P* > .05), indicating that rhEpo did not moderate any of the associations between age at assessment, sex, family SES, or gestational age and executive functions or processing speed. Results were similar if only complete cases were included in the analyses and if nonindependence in case of siblings was considered using a random-effects model. Also, the between-group differences remained similar when only those 190 children who had received rhEpo or placebo per protocol were included in the analyses (Figure 2).

**Table 2.** Descriptive Statistics of Executive Functions and Processing Speed Assessed for the EpoKids Study in Children Born Very Preterm Who Had Received rhEpo, Children Born Very Preterm Who Had Received a Placebo, and Typically Developing Term-Born Children^a^

Variables	Erythropoietin group (max n = 117)		Placebo group (max n = 97)		Term-born control group (max n = 167)	
	No.	Mean (SD)	No.	Mean (SD)	No.	Mean (SD)
Inhibition						
Stop-signal task (Stop-Signal Reaction Time; shorter = better), ms	80	245 (64)	66	248 (53)	155	235 (56)
D-KEFS CWIT (completion time; shorter = better), s	99	86.5 (22.3)	75	86.0 (23.0)	159	80.2 (22.0)
D-KEFS CWIT (No. errors; fewer = better), median (IQR)	98	0 (0-1)	75	0 (0-1)	158	0 (0-1)
Working memory						
TAP Working Memory (No. errors; fewer = better), median (IQR)^b^	89	11.0 (8-17)	66	11.0 (7-14.8)	160	8.0 (4-13)
Corsi Block Task (No. correct; more = better)^c^	101	15.6 (4.0)	75	15.6 (3.9)	166	17.6 (3.5)
WISC-IV Digit Span (No. correct; more = better)^c^	93	13.5 (2.5)	72	13.8 (2.8)	166	14.6 (3.3)
Cognitive flexibility						
D-KEFS TMT (completion time; shorter = better), s	85	121.1 (46.7)	66	112.1 (45.3)	157	94.8 (39.2)
TAP Flexibility (No. errors; fewer = better), median (IQR)	84	5 (2-9.3)	66	4.5 (1.3-9)	160	4 (2-9)
TAP Flexibility (reaction time; shorter = better), ms	84	1141 (244)	66	1141 (326)	160	1072 (294)
Fluency						
D-KEFS Design Fluency (No. correct; more = better)^d^	94	6.9 (2.4)	71	7.3 (2.6)	164	8.0 (2.7)
RWT Animals (No. correct; more = better)^d^	101	25.1 (7.6)	72	25.9 (7.0)	166	28.3 (8.0)
Planning						
D-KEFS Tower Task (total score; higher = better)	89	14.9 (3.2)	70	15.7 (3.1)	142	16.1 (2.6)
Executive functions in everyday life (T score; normative mean [SD]: 50 [10]; higher = more problems)						
BRIEF Global score	101	50.0 (9.9)	87	51.8 (10.3)	161	49.1 (8.7)
BRIEF Behavioral regulation	103	50.3 (10.5)	89	52.0 (10.4)	161	49.3 (9.1)
BRIEF Metacognition	101	49.9 (9.7)	87	51.6 (10.2)	162	49.1 (8.8)
Processing speed						
Stop-signal task (No-Signal Reaction Time; shorter = better), ms	80	682 (63.3)	66	676 (67)	155	668 (69)
WISC-IV Symbol Search (total score; higher = better)	91	22.7 (5.6)	71	23.1 (7.3)	163	25.7 (6.4)
WISC-IV Coding (total score; higher = better)	93	39.5 (10.1)	72	40.0 (10.4)	164	43.5 (11.7)

Abbreviations: BRIEF, Behavior Rating Inventory of Executive Functions^35^; CWIT, Color Word Interference Task; D-KEFS, Delis-Kaplan Executive Function System^28^; max, maximum; min, minimum; rhEpo, recombinant human erythropoietin; RWT, Regensburger Verbal Fluency Test (Regensburger Wortflüssigkeitstest)^34^; TAP, Test Battery for Attentional Performance (Testbatterie zur Aufmerksamkeitsprüfung)^31^; WISC-IV, Wechsler Intelligence Scale for Children, Fourth Edition, German version^32^.

^a^ Raw data (ie, completion time or reaction time, No. of errors, No. of correct items) are presented for the performance-based tasks because normative data are not available for all tasks. For parent-rated executive functions in everyday life (BRIEF), T-scores derived from normative data are presented.

^b^ Sum of omission and commission errors.

^c^ Total score forward and backward.

^d^ In 2 minutes.

**Figure 3.**
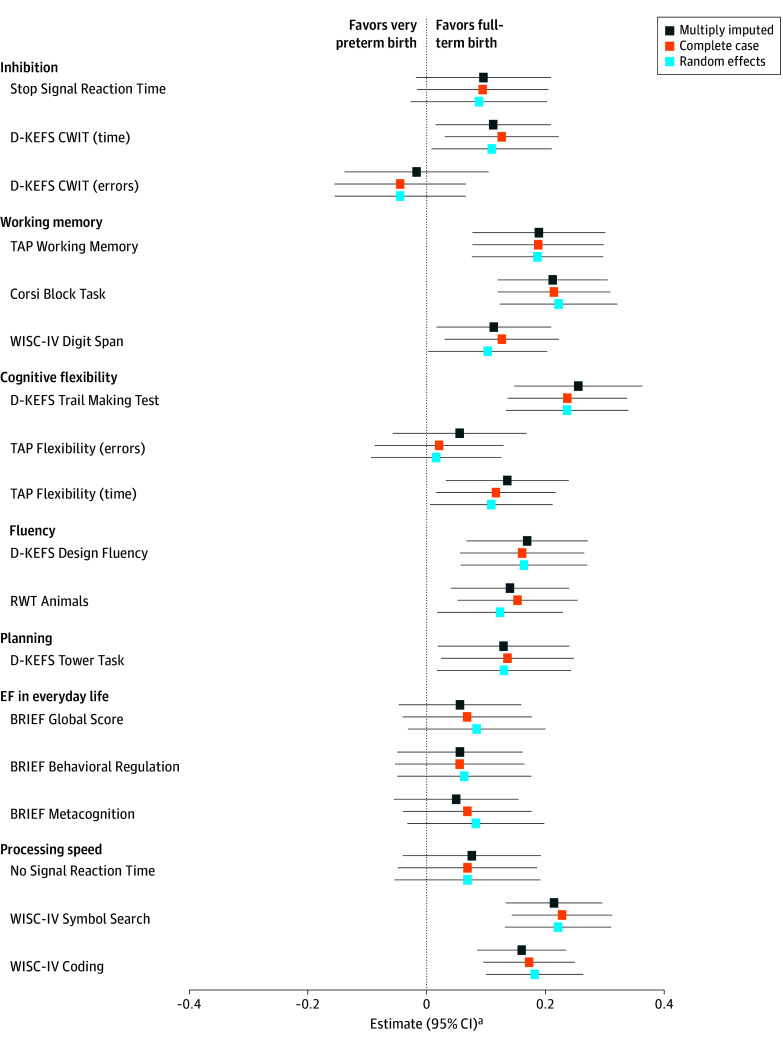
Group Differences in Executive Functions and Processing Speed Between Children Born Very Preterm and Term-Born Children ^a^Coefficients of multivariable linear regression models adjusted for age at assessment, sex, and family socioeconomic status. BRIEF indicates Behavior Rating Inventory of Executive Functions^35^; CWIT, Color Word Interference Task; D-KEFS, Delis-Kaplan Executive Function System^28^; RWT, Regensburger Verbal Fluency Test (Regensburger Wortflüssigkeitstest)^34^; TAP, Test Battery for Attentional Performance (Testbatterie zur Aufmerksamkeitsprüfung)^31^; WISC-IV, Wechsler Intelligence Scale for Children, Fourth Edition, German version^32^.

Irrespective of rhEpo or placebo allocation, children born very preterm had lower executive function and processing speed scores in at least 1 test in each domain compared with term-born peers (range of estimates: 0.112 to 0.255, 95% CIs did not include 0) (eTable 3 in Supplement 1). Executive functions in everyday life as rated by parents were not different between groups (Figure 3). The interactions between group (very preterm vs term-born) and age at assessment, sex, and family SES were not significant (all *P* > .05), except for the Corsi Block Task (interaction between group and family SES: *P* = .048).

## Discussion

In this long-term observational follow-up study to investigate the association between prophylactic early high-dose rhEpo administered to very preterm infants and their neurodevelopmental outcome at school age, no difference in executive functions and processing speed was found between those children who had received rhEpo and those who had received a placebo. These findings do not support the hypothesis of the EpoKids study that positive associations between early high-dose rhEpo and neurodevelopmental outcome become apparent as very preterm infants grow older and increasingly complex cognitive abilities emerge.

The Swiss EPO Neuroprotection Trial had found no improvements after rhEpo administration, neither in cognitive development at 2 years (primary outcome)^15^ nor in cognitive development or behavior and quality of life at 5 years (secondary outcome).^26,43^ These findings and those of other early childhood outcome studies (eg, Juul et al^14^) together with reports of improved brain development at term-equivalent age in a subset of the Swiss EPO Neuroprotection Trial cohort^16,17,18^ prompted the call for longer follow-up periods after neonatal interventions to determine whether beneficial effects become apparent later during development.^12,19,44^

The EpoKids study had set out to investigate this topic. Several issues now require consideration when interpreting the findings and searching for explanations for the lack of an association between rhEpo administration and better executive functions and processing speed. First, the 3 high-dose rhEpo administrations (3000 IU/kg) within the first 2 days of life may not have been sufficient to improve the long-term neurodevelopmental outcome, even though the dosage and timing had been determined from available experimental data when the Swiss EPO Neuroprotection Trial was initiated,^25^ and improved brain development has been reported in a subset of infants at term-equivalent age.^16,17,18^ Such an early, short-term high-dose administration may, however, disregard the secondary and tertiary phases of neonatal brain injury that extend weeks, months, and possibly years into postnatal life.^8,19,45,46,47^ In fact, erythropoietin has been suggested to be useful as a neuroprotective and as a neurorestorative intervention, and as such, requiring prolonged administration to be effective.^19^ Lending some support to this, a small trial, indeed, reported improved neurodevelopmental outcome through early school age in children born very preterm who had received erythropoiesis-stimulating agents through 35 weeks postconceptional age.^48,49,50^ In contrast, the large PENUT trial that complemented initial high-dose rhEpo with maintenance doses through 32 completed weeks of postmenstrual age neither found a beneficial effect at 22 to 26 months^14^ nor any evidence for improved brain development.^51,52^ Accordingly, further studies may be needed to investigate whether prolonged administration of rhEpo, potentially even beyond term-equivalent age, can improve long-term neurodevelopmental outcome after very preterm birth.

Second, the final EpoKids sample size fell short of the targeted one (ie, 214 vs 296 children^24^). This may have limited statistical power to identify actual group differences. However, no definite trend across the broad set of neuropsychological tests was apparent that may hint toward such a potential undetected association between rhEpo administration and better executive functions and processing speed.

Third, the cohort of children born very preterm may have been biased toward a high-performing subset, thus leaving little room for further improvement through a neuroprotective intervention. Indeed, as is common in longitudinal studies,^53^ those children who participated in the EpoKids study had better cognitive development in early childhood and came from a higher SES background than those who did not participate. Still, the EpoKids cohort experienced substantial problems in executive functions and processing speed compared with term-born peers: their performance was significantly lower in all domains except for everyday-life executive functions. In fact, in contemporary cohorts of children born very preterm, poor executive functions and slow processing speed are among the most frequent neurodevelopmental problems,^3,5^ often occurring in the absence of other impairments (eg, Wehrle et al^54^), and exerting negative cascade effects on other developmental domains such as academic achievement,^55,56,57,58^ behavior,^59,60,61^ or well-being and quality of life.^62,63^ Thus, the EpoKids cohort likely represents the neurodevelopmental profile of today’s children born very preterm.

Finally, rhEpo, by itself, may not effectively improve neurodevelopmental outcome after very preterm birth. While findings of a small trial suggest otherwise,^48,49,50^ the multitude of factors that impact development, including exposure to pre-, peri-, and postnatal environments, make this explanation the most probable one. On neonatal intensive care units, developmental care practices such as the Newborn Developmental Care and Assessment Program (NIDCAP^64^) and care approaches that evolve around the family (eg, family-centered or family-integrated care^65,66^) aim to optimize the environments and experiences to which the preterm infants and their families are exposed. Evidence shows that this, indeed, benefits the short-term and long-term neurodevelopment of the preterm infants and improves the mental health of parents.^67,68,69,70^ Furthermore, postdischarge developmental interventions have been found to benefit the motor and cognitive outcome^71^ and high family SES—commonly associated with favorable characteristics of the child’s upbringing, including parental warmth and positive parenting^72^—has even been shown to attenuate the impairing effect of neonatal brain injury on neurodevelopmental outcome.^73^ These findings convincingly illustrate that environments and experiences contribute to shaping a child’s neurodevelopment, and should, thus, be considered in the bundles of care,^74^ including pharmacological and nonpharmacological approaches that aim to support long-term outcome after very preterm birth.

### Strength and Limitations

As a clear strength, the EpoKids study applied validated neuropsychological tests and questionnaires to reliably characterize the executive function and processing speed profile of a large cohort of children born very preterm at school age, followed since birth, and added a group of term-born peers as contemporaneous comparison group. Although a number of limitations related to the size and composition of the sample have been discussed already, additional limitations of this study require consideration. As is usual in follow-up studies of neonatal clinical trials,^44^ the EpoKids assessment at school age was not part of the original study protocol to which parents had consented upon their child’s birth. Rather, families were recontacted and enrolled anew for the EpoKids study. While blinding remained intact, the distribution of several neonatal and developmental characteristics became imbalanced between the 2 treatment groups. Also, the Swiss EPO Neuroprotection Trial and, consequently the EpoKids study, did not enroll extremely preterm infants born before 26 weeks of gestation. This precludes conclusions about the long-term effect of early high-dose rhEpo in this particularly at-risk subgroup of very preterm patients.

## Conclusions

In this long-term, observational follow-up cohort study of a randomized clinical trial, there was no evidence of a positive association between early high-dose rhEpo and executive functions and processing speed in children born very preterm at school age. Likely, a comprehensive approach, including pharmacological and nonpharmacological prevention and intervention strategies, is needed to support these children’s neurodevelopmental outcomes across their lives.

## Notes

Supplement 1. **eTable 1.** Socio-Demographic, Perinatal and Neurodevelopmental Characteristics of Participating and Non-Participating Children Born Very Preterm **eTable2.** Group Differences in Executive Functions and Processing Speed Between Children Born Very Preterm Who Had Received High-Dose Recombinant Human Erythropoietin Shortly After Birth and Children Who Had Received a Placebo **eTable 3.** Group Differences in Executive Functions and Processing Speed Between Children Born Very Preterm and Term-Born Children
eReferences

Supplement 2.
Data Sharing Statement
