# Supplementary material for: Early High-Dose Erythropoietin and Cognitive Functions of School-Aged Children Born Very Preterm
Source: JAMA Netw Open. 2024 Sep 10;7(9):e2430043. doi: 10.1001/jamanetworkopen.2024.30043 (PMC11388032; doi:10.1001/jamanetworkopen.2024.30043)
Supplement: Supplement 1. — eTable 1. Socio-Demographic, Perinatal and Neurodevelopmental Characteristics of Participating and Non-Participating Children Born Very Preterm eTable2. Group Differences in Executive Functions and Processing Speed Between Children Born Very Preterm Who Had Received High-Dose Recombinant Human Erythropoietin Shortly After Birth and Children Who Had Received a Placebo eTable 3. Group Differences in Executive Functions and Processing Speed Between Children Born Very Preterm and Term-Born Children eReferences [file jamanetwopen-e2430043-s001.pdf]

Supplemental Online Content

Wehrle FM, Held U, Disselhoff V, et al. Early high-dose erythropoietin and cognitive functions of school-aged children born very preterm. *JAMA Netw Open.* 2024;7(9):e2430043. doi:10.1001/jamanetworkopen.2024.30043

eTable 1. Socio-Demographic, Perinatal and Neurodevelopmental Characteristics of Participating and Non-Participating Children Born Very Preterm

eTable2. Group Differences in Executive Functions and Processing Speed Between Children Born Very Preterm Who Had Received High-Dose Recombinant Human Erythropoietin Shortly After Birth and Children Who Had Received a Placebo

eTable 3. Group Differences in Executive Functions and Processing Speed Between Children Born Very Preterm and Term-Born Children

eReferences

This supplemental material has been provided by the authors to give readers additional information about their work.

© 2024 Wehrle FM et al. *JAMA Network Open.*

eTable 1. Socio-demographic, perinatal and neurodevelopmental characteristics of participating and non-participating children born very preterm.

	**EpoKids participants** (*n*=214)	**EpoKids non-participants** (*n*=151)	***SMD***[a]
**Socio-demographic characteristics**			
Sex (males, no. [%])	117 (54.7)	101 (66.9)	0.252
Family socio-economic status (*Md* [*IQR*])[b]	5 (4, 6)	6 (4, 8)	0.503
**Perinatal characteristics**			
Treatment allocation (no. [%] rhEpo)	117 (54.7)	74 (49)	0.114
Gestational age (in weeks, *M* [*SD*])	29.2 (1.7)	29.3 (1.6)	0.025
Birth weight (in grams, *M* [*SD*])	1200 (333)	1241 (351)	0.120
Head circumference at birth (in cm, *M* [*SD*])	26.9 (2.2)[c]	27.0 (2.2)[d]	0.048
Apgar at 5-minutes (*Md* [*IQR*])	8 (7, 9)[e]	8 (7, 9)[f]	0.103
**2-year outcome**[g]			
Mental Development Index (*M* [*SD*])	96.8 (16.2)	89.9 (17.1)	0.417

[a]Standardized Mean Difference (SMD) between the participants and non-participants of the EpoKids study, SMD < with 0.1 indicating balance between groups. [b]estimated from maternal education and paternal occupation assessed at birth, each rated on a 6-point scale and subsequently summed to form a scale ranging from 2 to 12, higher scores indicate lower family socio-economic status; for 36 children (18 participants, 18 non-participants) data was missing. [c]for two children born very preterm, this data was missing. [d]for one child born very preterm, this data was missing. [e]for one child born very preterm, this data was missing. [f]for five children born very preterm, this data was missing. [g]assessed with the Bayley Scales of Infant Development, second edition (BSID-II)[1].

© 2024 Wehrle FM et al. *JAMA Network Open.*

eTable2. Group differences in executive functions and processing speed between children born very preterm who had received high-dose recombinant human erythropoietin shortly after birth and children who had received a placebo.

	Estimate[a]	Lower 95%-CI	Upper 95%-CI
**Inhibition**			
Stop Signal Task (reaction time)	0.017	-0.139	0.172
D-KEFS CWIT (time)	-0.021	-0.162	0.119
D-KEFS CWIT (errors)	-0.016	-0.174	0.142
**Working memory**			
TAP working memory	-0.127	-0.294	0.039
Corsi Block Task	-0.018	-0.151	0.115
WISC-IV Digit Span	-0.086	-0.215	0.044
**Cognitive flexibility**			
D-KEFS TMT	-0.116	-0.266	0.034
TAP Flexibility (errors)	-0.036	-0.198	0.126
TAP Flexibility (time)	-0.033	-0.184	0.118
**Fluency**			
D-KEFS Design Fluency	-0.099	-0.253	0.055
RWT Animals	-0.057	-0.202	0.087
**Planning**			
D-KEFS Tower Task	-0.138	-0.290	0.013
**Executive functions in everyday life**			
BRIEF Global score	0.084	-0.049	0.218
BRIEF Behavioral regulation	0.077	-0.062	0.216
BRIEF Metacognition	0.081	-0.055	0.218
**Processing speed**			
Stop Signal Task (no reaction time)	-0.058	-0.221	0.104
WISC-IV Symbol Search	-0.067	-0.194	0.061
WISC-IV Coding	-0.059	-0.180	0.062

[a]Estimates are coefficients of multivariable linear regression models adjusted for age at assessment, sex, family socio-economic status and gestational age. Data was 50-fold multiply imputed to account for missingness. The missing data generating mechanism was considered to be missing at random, and the multiple imputation model included all outcomes, the grouping variable (i.e., rhEpo, placebo, control group), and the adjustment variables age at assessment, sex, and family SES.
BRIEF: Behaviour Rating Inventory of Executive Functions[2]. CI: Confidence interval. CWIT: Color Word Interference Task. D-KEFS: Delis-Kaplan Executive Function System[3]. RWT: Regensburger Verbal Fluency Test (‘Regensburger Wortflüssigkeitstest’[4]). TAP: Test Battery for Attentional Performance (‘Testbatterie zur Aufmerksamkeitsprüfung’[5]). WISC-IV: Wechsler Intelligence Scale for Children, Fourth Edition, German version[6].

© 2024 Wehrle FM et al. *JAMA Network Open.*

eTable 3. Group differences in executive functions and processing speed between children born very preterm and term-born children.

	Estimate[a]	Lower 95%-CI	Upper 95%-CI
**Inhibition**			
Stop Signal Task (reaction time)	0.095	-0.019	0.209
D-KEFS CWIT (time)	0.112	0.015	0.209
D-KEFS CWIT (errors)	-0.018	-0.139	0.104
**Working memory**			
TAP working memory	0.189	0.077	0.301
Corsi Block Task	0.212	0.119	0.305
WISC-IV Digit Span	0.113	0.016	0.210
**Cognitive flexibility**			
D-KEFS TMT	0.255	0.147	0.364
TAP Flexibility (errors)	0.055	-0.058	0.168
TAP Flexibility (time)	0.136	0.032	0.240
**Fluency**			
D-KEFS Design Fluency	0.169	0.067	0.271
RWT Animals	0.140	0.040	0.240
**Planning**			
D-KEFS Tower Task	0.129	0.018	0.240
**Executive functions in everyday life**			
BRIEF Global score	0.056	-0.048	0.159
BRIEF Behavioral regulation	0.056	-0.050	0.161
BRIEF Metacognition	0.049	-0.056	0.154
**Processing speed**			
Stop Signal Task (no reaction time)	0.076	-0.041	0.192
WISC-IV Symbol Search	0.214	0.133	0.296
WISC-IV Coding	0.160	0.085	0.235

[a]Estimates are coefficients of multivariable linear regression models adjusted for age at assessment, sex and family socio-economic status. Data was 50-fold multiply imputed to account for missingness. The missing data generating mechanism was considered to be missing at random, and the multiple imputation model included all outcomes, the grouping variable (i.e., rhEpo, placebo, control group), and the adjustment variables age at assessment, sex, and family SES.

BRIEF: Behaviour Rating Inventory of Executive Functions[2]. CI: Confidence interval. CWIT: Color Word Interference Task. D-KEFS: Delis-Kaplan Executive Function System[3]. RWT: Regensburger Verbal Fluency Test (‘Regensburger Wortflüssigkeitstest’[4]). TAP: Test Battery for Attentional Performance (‘Testbatterie zur Aufmerksamkeitsprüfung’[5]). WISC-IV: Wechsler Intelligence Scale for Children, Fourth Edition, German version[6].

© 2024 Wehrle FM et al. *JAMA Network Open.*

eReferences

1. Reuner G, Rosenkranz J, Pietz J, Horn R. Bayley Scales of Infant Development, Second Edition (Bayley II)—Deutsche Fassung. In. Frankfurt am Main, Germany: Pearson Assessment; 2007.
2. Drechsler R, Steinhausen H-C. BRIEF: Verhaltensinventar zur Beurteilung exekutiver Funktionen [BRIEF: Behaviour Rating Inventory of Executive Functions]. In. Bern, Switzerland: Huber; 2013.
3. Delis D, Kaplan E, Kramer J. Delis-Kaplan Executive Function System (D-KEFS). In: Psychological Corporation; 2001.
4. Aschenbrenner S, Tucha O, Lange K. Regensburger Wortflüssigkeits-Test (RWT) [Regensburger Verbal Fluency Test (RWT)]. In. Göttingen, Germany: Hogrefe; 2000.
5. Zimmermann P, Fimm B. Testbatterie zur Aufmerksamkeitsprüfung [Test Battery for Attentional Performance]. In. Herzogenrath, Germany1993.
6. Petermann F, Petermann U. Hamburg-Wechsler Intelligenztest für Kinder IV (HAWIK-IV) [Hamburg-Wechsler Intelligence Scale for Children IV (WISC-IV)]. In. Bern, Switzerland: Huber; 2006.

© 2024 Wehrle FM et al. *JAMA Network Open*.
