# Supplementary material for: Early High-Dose Erythropoietin and Cognitive Functions of School-Aged Children Born Very Preterm
Source: JAMA Netw Open. 2024 Sep 10;7(9):e2430043. doi: 10.1001/jamanetworkopen.2024.30043 (PMC11388032; doi:10.1001/jamanetworkopen.2024.30043)
Supplement: Supplement 2. — Data Sharing Statement [file jamanetwopen-e2430043-s002.pdf]

Data Sharing Statement

Wehrle. Early High-Dose Erythropoietin and Cognitive Functions of School-Aged Children Born Very Preterm. *JAMA Netw Open*. Published September 06, 2024.
doi:10.1001/jamanetworkopen.2024.30043

Data
Data available: No

Additional Information
Explanation for why data not available: The approval granted by the ethical committee of the Canton of Zurich, Switzerland for the EpoKids study (Basec No. 2017-00521) does not allow the publication of the raw data online. If readers would like to re-analyse the data set for other purposes, additional ethical approval will be required for each individual user and purpose. The authors are happy to support additional ethical approval applications from researchers for access to this data set (contact: cornelia.hagmann@kispi.uzh.ch)
